# Beliefs about antiretroviral therapy and their association with adherence in young people living with perinatal HIV in England: a cross-sectional analysis

**DOI:** 10.1080/09540121.2023.2300984

**Published:** 2024-01-25

**Authors:** Iona White, Ali Judd, Hannah Castro, Elizabeth Chappell

**Affiliations:** MRC Clinical Trials Unit at UCL, UCL, London, UK

**Keywords:** beliefs, adherence, antiretroviral therapy, HIV, young people

## Abstract

This cross-sectional analysis aimed to describe beliefs about antiretroviral therapy (ART) in young people living with perinatal HIV (PHIV) in England, and the association between these beliefs and adherence to ART. The Beliefs About Medicine Questionnaire (Highly Active Antiretroviral Therapy version), was used to measure participants’ beliefs in the necessity of (“Necessity score”) and concerns regarding (“Concerns score”) ART. Participants were classified as having high/low total scores using midpoints of the score scales. Associations between beliefs and being Last Month Adherent (LMA; self-reported not missing more than 2 consecutive ART doses in the month prior to the interview) were analysed using logistic regression, adjusting for sociodemographic, clinical, and psychosocial variables. Of 247 PHIV (median age = 18.6 years), 158 (64%) were LMA. 224 (91%) had a high Necessity score and 54 (22%) a high Concerns score. There was no association between high Necessity score and LMA in multivariable analysis (adjusted odds ratio (aOR) = 1.34, 95% confidence interval (CI) = 0.34–5.28, *p* = 0.679); however, high Concerns score was independently associated with a reduced odds of being LMA (aOR = 0.19, CI = 0.07–0.47, *p* < 0.001). Interventions to address the concerns young people living with PHIV have about ART should be explored as a strategy to improve their adherence.

## Introduction

Suboptimal antiretroviral therapy (ART) adherence among young people (adolescents and young adults) living with HIV presents a barrier to ending the AIDS epidemic by 2030 (The Joint United Nations Programme on HIV/AIDS et al., 2022). Virological suppression has multiple benefits including reduced risks of disease progression, mortality and viral transmission (Bello et al., 2016; Elvstam et al., 2021; Rodger et al., 2016). In 2019, virological suppression in the UK was lowest among people aged 15–24 years (91%) and those who acquired HIV vertically (89%) (Public Health England, 2020). Newer ART regimens require higher levels of adherence (at least 80–90%), depending on regimen type, to achieve virological suppression (Byrd et al., 2019). However, a meta-analysis of 50 studies of adolescents with HIV reported only 62% were acceptably adherent to therapy (95% confidence interval (CI) = 57.1–67.6%) with the lowest adherence observed in North America and Europe (Kim et al., 2014).

Several reasons for poor adherence in young people living with perinatal HIV (PHIV) have been identified including stigma, diagnosed depression, the bitter taste of protease inhibitor (PI)-based regimens and treatment fatigue due to having taken ART since infancy (Fields et al., 2017; Judd et al., 2020; Kacanek et al., 2019).

The beliefs patients hold about their medication have also been investigated as potential reasons why some people with chronic conditions (including HIV) are non-adherent (Al Bawab et al., 2021; Cea-Calvo et al., 2020; Shahin et al., 2020). The Beliefs about Medicines Questionnaire – Specific (BMQ-Specific) was developed to measure patients’ beliefs regarding the necessity of, and concerns about the negative effects of, their prescribed medication (Horne et al., 1999). The questionnaire was subsequently adapted to produce the Beliefs about Medicines Questionnaire – Highly Active Antiretroviral Therapy version (BMQ-HAART) to assess beliefs regarding ART in people living with HIV (Horne et al., 2004).

Three studies have investigated the relationship between beliefs about ART and different adherence measures in young people living with HIV. Kang et al. used an adapted version of the BMQ-Specific questionnaire to measure beliefs about ART in 89 young people living with PHIV in Thailand and found no significant association with adherence (measured as no self-reported missed doses in the past 7 days, receiving a rating of “very good” or “good” adherence from the caregiver, and having latest viral load < 1000 copies/ml) (Kang et al., 2015). Garvie et al. used the Beliefs About Medicine Scale (BAMS) to measure beliefs about ART in 20 young people living with behaviourally acquired HIV in America and found that a higher Perceived Threat of Illness (general health beliefs) subscore and a higher total score were associated with better adherence to a Directly Observed Therapy intervention (Garvie et al., 2011; Riekert & Drotar, 2002). Navarra et al. also used BAMS to measure beliefs about ART in 50 young people living with either perinatal or behaviourally acquired HIV in America and reported that a higher Positive Outcome Expectancy (the belief that medicine will make a person well) subscore was associated with increased odds of 100% adherence (measured as a self-reported 3-day adherence estimate) (Navarra et al., 2014). However, these studies may have limited generalisability due to small sample sizes, the populations included and different outcome measures.

The Adolescents and Adults Living with Perinatal HIV (AALPHI) was a prospective cohort study which included 296 APHIV (aged 13–21 years) living in England. Participants underwent two interviews over a 5-year period to investigate clinical and psychosocial outcomes (Judd et al., 2016). Two previous analyses of factors affecting medicine adherence using data from the first interview have been conducted in this cohort (Hawkins et al., 2016; Judd et al., 2020). However, neither analysis investigated the relationship between beliefs about ART and adherence using BMQ-HAART data collected at the second interview. As the published literature to date on young people with HIV is limited to the three studies described previously, our study provides an opportunity to fill an important gap in the evidence base.

Understanding the beliefs about ART held by PHIV young people in England and their association with adherence is important to inform HIV service delivery and the development of interventions to improve adherence to ART. In this study, we explore the association between adherence to ART and beliefs about medicine in the AALPHI cohort.

## Methods

AALPHI was a prospective study evaluating the impact of HIV infection and ART exposure on young people living with PHIV in England and comparing outcomes to those for young people not living with HIV, across several research areas. Participants were enrolled from HIV clinics and community organisations in England and undertook first interviews between 2013 and 2015, and second interviews between 2015 and 2017. This cross-sectional analysis included only young people in AALPHI living with PHIV and utilised data collected from second interviews.

Eligibility criteria at enrolment to the study included age 13–21 years, a history of paediatric HIV care in the UK, having lived in the UK for at least six months and the ability to speak and understand English (Judd et al., 2016). All participants living with PHIV had known their HIV status for at least 6 months and were all included in the national UK and Ireland Collaborative HIV Paediatric Study (CHIPS) (Collins et al., 2017). Full ethical approval was received from the Leicester Research Ethics Committee (reference 12/EM/0012). The requirement for parental consent was waived for participants less than 18 years of age if it was deemed by the study research nurses that the participant had the capacity to provide written informed consent. Otherwise, the young person provided written assent and a parent or guardian provided written informed consent.

### Procedures

Data were collected via a 2-hour face-to-face interview with a study research nurse. This included a Computer-Assisted Self-Interview (CASI) in which the participant answered questions on adherence and completed the BMQ-HAART questionnaire. Due to the potential for interview questions to raise sensitive subjects, only participants who had come to terms with their diagnosis were approached to participate. If the participant became upset, the study research nurse would pause or stop the interview as appropriate to discuss any issues arising. Safeguarding protocols were in place and appropriate referral pathways were established where required.

### Beliefs about medicine

Participants’ beliefs about ART were measured using the BMQ-HAART (Horne et al., 2004) which comprises an 8-item Necessity subscale assessing participants’ beliefs about the necessity of their prescribed ART medication (each item scored 1–5, total range of 8–40) and an 11-item Concerns subscale assessing their concerns about the potential adverse consequences of taking their ART medication (each item scored 1–5, total range 11–55). Higher scores indicated stronger necessity or concerns beliefs (Horne et al., 2004) (see Appendix A). Cronbach’s Alpha (*α*) was used as a measure of the internal consistency of the items in each subscale and was acceptable (≥0.70) for both the Necessity (*α* = 0.76) and Concerns subscales (*α* = 0.77).

### Adherence

Two self-reported adherence outcomes were collected in order to compare adherence over two different recall periods: “Last Month Adherence” (did not miss more than two ART doses in a row the month prior to interview) and “3-day Adherence” (did not miss any doses in the three days prior to interview).

### Viral load

The nearest viral load measurement within 6 months before or after the interview date was used in the analysis. Viral suppression was defined as a viral load <50 copies/ml.

### Statistical analysis

Participants were included in the analysis if they had completed both adherence questions, the BMQ-HAART questionnaire and were taking ART at the time of interview. Analyses were conducted using Stata/MP version 17 (Stata Corp, College Station, TX).

### Descriptive statistics

Mean Necessity and mean Concerns scores for each participant were calculated by dividing the total score for each BMQ-HAART subscale for each participant by the number of items in the subscale. The Necessity-Concerns Differential (NCD) was calculated by subtracting the mean Concerns score from the mean Necessity score (Horne et al., 1999). A positive NCD indicates that belief in the necessity of ART outweighs the participant’s concerns about taking ART (Horne & Weinman, 1999). Participants were classified as having high/low Necessity and high/low Concerns scores using the midpoints of the total score scales (>/≤24 and >/≤33 respectively) and were divided into four attitudinal groups: Sceptical (low necessity, high concerns), Indifferent (low necessity, low concerns), Ambivalent (high necessity, high concerns) and Accepting (high necessity, low concerns) (Kosse et al., 2020).

Participants’ sociodemographic, clinical, and psychosocial characteristics were summarised as median and interquartile ranges (IQR) for continuous variables, and frequencies (%) for categorical variables. Characteristics were compared by high/low Necessity and high/low Concerns scores, using Chi-squared tests or Fisher’s exact tests for proportions (as appropriate given the number of cells with an expected value of five or greater) and Wilcoxon rank sum tests for medians. A *p*-value <0.05 was considered statistically significant.

### Regression modelling

Logistic regression models were constructed to assess the relationship between beliefs about ART and Last Month Adherence. The Last Month Adherence measure was selected as the dependent variable for all models as studies suggest that self-reported adherence measures with longer recall periods are more accurate (Farley et al., 2008; Lu et al., 2008). Complete-case analysis was carried out for the regression models, as missing data were assumed to be missing completely at random and there appeared to be no systematic differences between complete and incomplete cases (Hughes et al., 2019).

Three types of logistic regression model were constructed.

*Model 1* – Univariable regression models to explore the association between being Last Month Adherent (LMA) and each of high/low Necessity score and high/low Concerns score, and the following sociodemographic/clinical/psychosocial variables: sex (male vs female), age at interview (years), ethnicity (Black vs non-Black), birthplace (born outside the UK vs born in UK), living situation (housing association or council housing/flats vs other), occupation (in education vs other), parental vital status (one/both parents died vs both parents alive), age at ART initiation (years), years since ART initiation, total number of tablets taken per day (1 vs ≥ 2), type of ART regimen (PI-based vs other), CD4 count (nearest measurement within 6 months before or after the interview), Centers for Disease Control and Prevention (CDC) stage (Stage N/A/B vs Stage C; (Centers for Disease Control and Prevention, 1994)), type of care at interview (Adolescent/Adult vs Paediatric), heath-related quality of life (QoL) (EuroQol 5-Dimension 5-level (EQ5D) index score and EuroQol-Visual Analogue Scale (EQ-VAS) score (see Appendix A)), self-esteem (Rosenberg Self-Esteem Scale (SES) (see Appendix A)), and coping (Adolescent Coping Scale – 2nd Edition (ACS-2) Short Form subscale score (Productive Coping Usage, Productive Coping Helpfulness, Non-productive Coping Usage and Non-Productive Coping Helpfulness) (see Appendix A)).

*Model 2* – separate bivariable models including high/low Necessity and high/low Concerns scores alongside each variable (listed above) in turn, to assess the effect of each on the association between beliefs and adherence.

*Model 3* – a multivariable model to obtain an adjusted OR of being LMA for high/low Necessity and high/low Concerns scores when controlling for all sociodemographic, clinical, and psychosocial variables simultaneously. Type of care and occupation were found to be highly correlated with age at interview and were excluded from the multivariable model due to collinearity concerns (Cramer’s *V* ≥ 0.5). Similarly, age at ART initiation and type of ART regimen were found to be highly correlated with years since ART initiation and total number of tablets taken per day, respectively, and were also excluded from the multivariable model. Otherwise, variables were included regardless of the *p*-values attained in Models 1 and 2.

All models were also constructed with VL <50 copies/ml as the dependent variable to assess whether beliefs about ART were associated with suppressed viral load.

## Results

### Participant demographics

A total of 256 participants answered both adherence questions. Of these, seven were excluded as CHIPS data indicated that they were not on ART at the time of the interview. Two were excluded as they had incomplete BMQ-HAART data. Therefore, 247 participants were included in the analysis, for whom characteristics are summarised in Table 1.

The median age of participants was 18.6 years (IQR 17.0, 20.9). 146 (59%) were female, 216 (87%) were Black and 142 (57%) were born outside the UK. Approximately half of the young people were living in housing association or council housing/flats (120 (49%)) and approximately three-quarters were in education (189 (77%)). One or both parents of 95 (41% of 230) young people had died. 198 (80%) participants were prescribed 2 or more ART tablets per day and approximately half (1136 (55%)) were taking PI-based regimens. 163 (70% of 233) had a VL <50 copies/ml. 132 (53%) of young people had transitioned from paediatric to adolescent/adult care.

### Adherence and BMQ-HAART data

**Figure 1:**
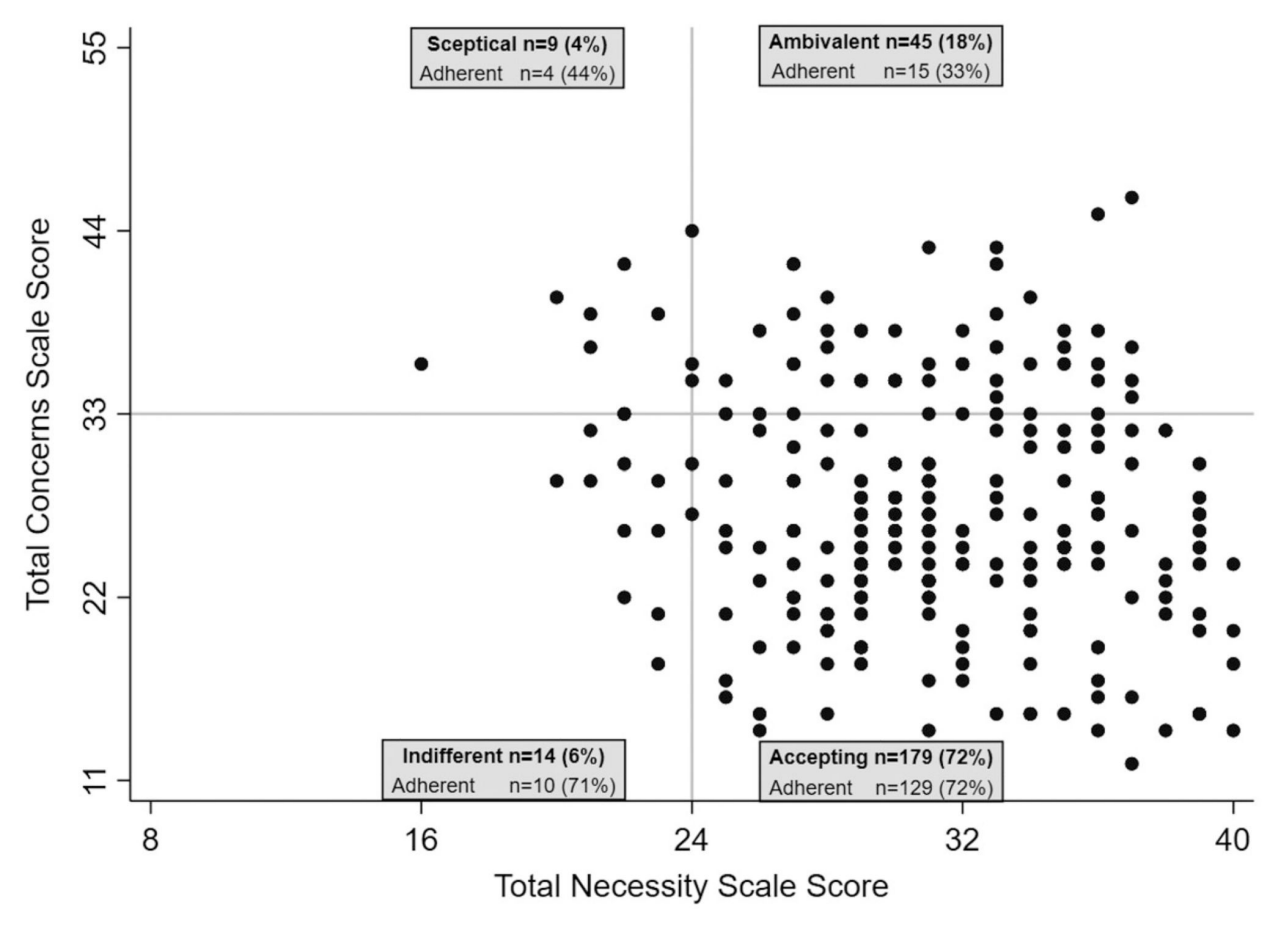
Scatter plot of BMQ-HAART total Necessity and Concerns scores (*n* = 247) divided into four attitudinal groups (Sceptical = Total Necessity score ≤24 and Total Concerns Score >33; Indifferent = Total Necessity score ≤24 and Total Concerns Score ≤33; Ambivalent = Total Necessity Score >24 and Total Concerns Score >33; Accepting = Total Necessity Score >24 and Total Concerns Score ≤33) and *n* (%) of participants in each group who did not miss more than two doses of antiretroviral therapy in the month prior to interview (Last Month Adherent). BMQ-HAART = Beliefs About Medicine Questionnaire – Highly Active Antiretroviral Therapy version.

172 (70%) participants were classed as 3-day Adherent and 158 (64%) as Last Month Adherent. The median BMQ-HAART Necessity mean score was 3.9 (IQR 3.5, 4.4) and the median Concerns mean score was 2.5 (IQR 2.1, 3.0). 224 (91%) participants had a high Necessity score and 54 (22%) had a high Concerns score. Participants generally had stronger necessity than concerns beliefs with a median NCD of 1.4 (IQR 0.7, 2.0). Approximately three-quarters of young people were classed as “Accepting” of ART (179 (72%)), with 45 (18%) “Ambivalent”, and only 14 (6%) and 9 (4%) “Indifferent” and “Sceptical” respectively (Figure 1). Participants classed as “Ambivalent” had the lowest prevalence of Last Month Adherence (15 (33%)) whereas “Accepting” participants had the highest prevalence (129 (72%)).

A similar proportion of participants with a high Necessity score were LMA (144 (64%)) compared to those with a low Necessity score (14 (61%), *p* = 0.745). A higher proportion of participants with a high Necessity score had one or both parents who had died compared to those with a low Necessity score (93 (45%) vs 2 (10%), *p* = 0.002). They also had a higher median Productive Coping Usage score (66% (IQR 59%, 74%) vs 60% (54%, 68%), *p* = 0.010) (Table 1).

A lower proportion of those with high Concerns scores were LMA than those with low Concerns scores (19 (35%) vs 139 (72%), *p* < 0.001). A higher proportion of participants with high Concerns scores were not in education or employment compared to those with low Concerns scores (9 (17%) vs 9 (5%), *p* = 0.013), and a lower proportion with high Concerns scores were taking an NNRTI-based regimen (8 (15%) vs 62 (32%), *p* = 0.018). Fewer participants with high Concerns scores had a VL <50 copies/ml (28 (53%) vs 135 (75%), *p* = 0.002), and this group had a lower median CD4 count (500 (IQR 363, 683) cells/mm^3^ vs 636 (472, 849) cells/mm^3^, *p* = 0.008). Health-related QoL was also lower in those with high Concerns scores (median EQ5D Index Score = 0.87 (IQR 0.73, 1.00) vs 0.94 (0.86, 1.00), *p* < 0.001; median EQ-VAS Score = 69 (50, 80) vs 80 (69, 90), *p* < 0.001), as was the median Rosenberg self-esteem score (18 (15, 22) vs 20 (16, 23), *p* = 0.011). Median Non-productive Coping Usage scores were higher in young people with high Concerns scores than low Concerns scores (60% (IQR 48%, 70%) vs 53% (45%, 65%), *p* = 0.009) as were Non-productive Coping Helpfulness scores (43% (38%, 55%) vs 40% (33%, 48%), *p* = 0.022) (Table 1).

### Regression analysis

The results of Models 1–3 are presented in Table 2 and Appendices B–E. A high Necessity score was not associated with being LMA in the univariable model (Model 1: odds ratio (OR) = 1.16, 95% CI = 0.48–2.79, *p* = 0.745), after adjustment for high/low Concerns score only (Model 2 adjusted OR (aOR) = 0.84, 95% CI = 0.32–2.18, *p* = 0.718) or when adjusting for high/low Concerns score and all the sociodemographic, clinical and psychosocial variables simultaneously (Model 3: aOR = 1.34, 95% CI = 0.34–5.28, *p* = 0.679). There was also no association between a high Necessity score and suppressed viral load (Model 1: OR = 1.18, 95% CI = 0.46–3.07, *p* = 0.730; Model 2: aOR = 0.94, 95% CI = 0.35–2.53, *p* = 0.902, Model 3: aOR = 1.42, 95% CI = 0.36–5.67, *p* = 0.615).

A high Concerns score significantly reduced the odds of being LMA in the univariable model (Model 1: OR = 0.21, 95% CI = 0.11–0.40, *p* < 0.001). This association remained significant when adjusting for a high Necessity score alone (Model 2: aOR = 0.21, 95% CI = 0.11–0.40, *p* < 0.001), with any of the other factors individually (*p* < 0.001 in all models (Appendix C)), and also in the fully adjusted model (Model 3: aOR = 0.19, 95% CI = 0.07–0.47, *p* < 0.001). The odds ratio was similar across all three models. Having a high Concerns score was also associated with lower odds of virological suppression in the univariable model and when adjusting for a high Necessity score (Model 1: OR = 0.37, 95% CI = 0.20–0.71, *p* = 0.002; Model 2: aOR = 0.37, 95% CI = 0.19–0.71, *p* = 0.003). However, the association was no longer statistically significant in the fully adjusted model (Model 3: 0.64, 95% CI = 0.25–1.52, *p* = 0.293).

Higher Non-productive Coping Usage score was borderline significantly associated with reduced odds of being LMA in the fully adjusted model (Model 3: aOR = 0.96, 95% CI = 0.93–1.00, *p* = 0.049), but there was no association with virological suppression (Model 3: aOR = 1.00, 95% CI = 0.97–1.04, *p* = 0.837) (Appendix E).

## Discussion

This study investigated the association between beliefs about ART and last month adherence in 247 young people with perinatal HIV in England. Approximately two-thirds of young people (158 (64%)) were Last Month Adherent, the majority (224 (91%)) had high beliefs about the necessity of their ART medication and approximately three-quarters (193 (78%)) had low concerns about the potential consequences of taking it. Having a high Concerns score was associated with lower odds of being Last Month Adherent in both a univariable model and when adjusted for sociodemographic, clinical and psychosocial variables (aOR = 0.19, 95% CI = 0.07–0.47, *p* < 0.001). Whereas, having a high Necessity score was not associated with Last Month Adherence in any model. There was also no association between high/low Necessity or high/low Concerns scores and viral suppression in the multivariable models.

Reported adherence at this interview was slightly lower compared to the first AALPHI interview, as was viral suppression (Last Month Adherent = 69%, 3-day Adherent = 73%, viral load <50 copies/ml = 76% in the first interview) (Judd et al., 2020). However, the adherence prevalence reported at both interviews was similar to estimates reported in previous studies in high-income countries, which ranged from 43% to 86% using various definitions of adherence (Bucek et al., 2018; Closson et al., 2019; Kim et al., 2014; Navarra et al., 2014).

In our study, participants generally had high mean Necessity item scores, low mean Concerns item scores and their beliefs in the necessity of ART outweighed their concerns. Approximately three-quarters of young people (179 (72%)) were classed as being “Accepting” of their medication (high necessity, low concerns) which is substantially higher than studies in 243 Dutch young adults with asthma (40%) and 112 Canadian young adults with inflammatory bowel disease (20%) (Fu et al., 2017; Kosse et al., 2020). Young people living with perinatal HIV in the AALPHI cohort may strongly believe in the necessity of ART as many are survivors of the pre-HAART era or were born just after and, on average, were 7.5 years old at ART initiation. Additionally, a large proportion of participants with high Necessity scores had experienced the death of one or both parents, potentially from HIV/AIDS. Therefore, lack of access to effective treatment at the start of life, and awareness of the potentially fatal consequences of their condition, may explain these beliefs.

A higher proportion of participants were classified as “Ambivalent” (high necessity, high concerns) and “Sceptical” (low necessity, high concerns) compared to Dutch young adults with asthma (18% vs 6% Ambivalent and 4% vs 1% Sceptical respectively) (Kosse et al., 2020). This difference may be due to the nature of the two conditions; HIV, as an infection, may be subject to a greater degree of stigma than asthma and therefore young people with perinatal HIV may be worried that taking ART in front of others may lead to unintended disclosure of their HIV status (Calabrese et al., 2012).

In this study, strong concerns regarding ART were independently associated with reduced odds of being Last Month Adherent in young people living with perinatal HIV when adjusting for other variables, while no association with beliefs in the necessity of ART was observed. These results are consistent with studies in adults living with HIV which reported a significant relationship between stronger concerns about ART and lower adherence (Batchelder et al., 2014; Horne et al., 2004; Mitzel et al., 2021). The only other study (using a version of the BMQ-Original) investigating associations between beliefs about ART and adherence in 89 young people living with perinatal HIV (median age 15 years) in Thailand, found no association between beliefs about ART and adherence. However, direct comparison to the study is difficult as it did not analyse the Necessity and Concerns scores separately. Additionally, most of the young people lived in orphanages where medication-taking was supervised, meaning that ART adherence was perhaps less of a personal choice than for young people in the AALPHI cohort (Kang et al., 2015). Other studies conducted on young people with HIV, measuring beliefs using the Beliefs About Medicine Scale, found that more positive beliefs about ART were associated with better adherence (Garvie et al., 2011; Navarra et al., 2014). However, both samples were small and contained some or all young people who acquired HIV other than perinatally, and the relationship between beliefs and adherence may differ by mode of HIV acquisition. These and the present analysis were conducted in samples where a majority held strong beliefs in the necessity of ART, and therefore limited variability may have reduced the ability to detect a significant association between necessity beliefs and adherence.

Abongomera et al. (2017) used a modified version of the BMQ-Specific in parents/carers of 271 children living with HIV (median age 2.8 years) initiating ART in sub-Saharan Africa and found that a higher Necessity-Concerns Differential score was strongly associated with better adherence measured by a Medication Event Monitoring System Cap and, at certain time periods, viral suppression. While the AALPHI cohort was older in age and therefore would likely take more responsibility for their medication-taking, it would nevertheless be interesting to explore the association between the beliefs of their parents/carers and ART adherence in young people with perinatal HIV, as well as associations with parents’/carers’ own adherence.

Our study has several limitations. Firstly, there is no “gold standard” measure of ART adherence. The Last Month Adherence measure was selected over 3-day Adherence as measures with longer recall periods are more reliable (Farley et al., 2008; Lu et al., 2008). However, the self-reported nature of outcomes used means that they may be subject to recall bias. Both adherence outcomes and the BMQ-HAART responses may also be affected by social desirability bias leading to overreporting of adherence and favourable beliefs about ART (Bangsberg, 2006). However, the likelihood of this was reduced as adherence and BMQ-HAART data were collected using Computer-Assisted Self-Interview with non-judgemental wording of questions. Also, similar adherence questions have been validated against viral load in previous studies in children and young people living with HIV (Scott et al., 2018). However, in the present study, although an association between high Concerns beliefs and reduced odds of being Last Month Adherent was observed, higher Concerns beliefs were not associated with reduced odds of viral suppression in the multivariable model. This difference may be because other factors aside from poor recent adherence may contribute to young people living with perinatal HIV having a viral load ≥50 copies/ml, for example, existing drug resistance mutations (Koay et al., 2021). In addition, the viral load measurements analysed in this study were taken within 6 months before or after the date of the interview and therefore, the participant’s beliefs about ART may have differed during this time-period.

Secondly, as the BMQ-HAART data were only collected at one AALPHI interview (to reduce the burden on participants, certain assessments were not conducted at every interview), the cross-sectional nature of the analysis means one cannot ascertain whether having stronger concerns about ART leads to lower adherence or low adherence causes stronger concerns beliefs, nor whether the strength of association fluctuates over time.

Thirdly, due to missing data issues and the use of complete-case analysis in the regression models, Model 3 only contained 73% of participants with complete beliefs and adherence data, potentially leading to biased estimates if the data were not missing completely at random.

Finally, as the interviews were conducted between 2015 and 2017, the findings may not be relevant to the current population of young people living with perinatal HIV in England. Since both AALPHI interviews were conducted, the use of new ART classes, such as integrase inhibitors, with higher efficacy and lower risk of treatment discontinuation, have become widespread which could have positively impacted patient adherence and beliefs in the necessity of ART (World Health Organization, 2019). However, the outbreak of the COVID-19 pandemic in 2020 may have had a particularly negative impact on people living with HIV including their access to treatment and mental health; as well as highlighting the widespread misinformation regarding, and public distrust in, science and medicine that has existed in recent years (Chenneville et al., 2020; Mian & Khan, 2020). It is unknown whether these factors have negatively impacted the beliefs that young people with perinatal HIV hold now about ART or their adherence.

In conclusion, in our study, while approximately two-thirds of young people with perinatal HIV were acceptably adherent to ART, some may require adherence support. While most participants were “Accepting” of ART, strong concerns beliefs were independently associated with reduced ART adherence. These findings could inform the development of tailored adherence interventions for young people with perinatal HIV to address their concerns about ART and methods of coping with them. Future research could use the BMQ-HAART in new perinatal HIV studies to expand the evidence base, and young people’s concerns could be investigated further through qualitative research.

## Supplementary Material

Appendices

## Figures and Tables

**Table 1:** Sociodemographic, clinical, and psychosocial characteristics of the total sample and disaggregated by BMQ-HAART high/low total Necessity score and high/low total Concerns score.

Characteristics	Total *n* = 247	High total Necessity score *n* = 224	Low total Necessity score *n* = 23	*p*- Value^b^	High total Concerns score *n* = 54	Low total Concerns score *n* = 193	*p*- value^b^
**Sex, *n* (%)** (vs Male)							
Female	146 (59)	133 (59)	13 (57)	0.791	33 (61)	113 (56)	0.735
**Age at interview (years), Median [IQR]**	18.6 [17.0, 20.9]	18.6 [17.0, 21.0]	18.4 [17.0, 20.8]	0.696	19.3 [17.7, 21.1]	18.4 [16.8, 20.8]	0.118
**Ethnicity, *n* (%)** (vs Non-Black)							
Black	216 (87)	195 (87)	21 (91)	0.748	50 (93)	166 (86)	0.197
**Birthplace, *n* (%)**(vs Born in UK)							
Born outside UK	142 (57)	130 (58)	12 (52)	0.588	28 (52)	114 (59)	0.343
**Living situation at time of interview, *n* (%)**							
Family own/rent house/flat	90 (36)	85 (38)	5 (22)	0.203	17 (31)	73 (38)	0.510
Housing association/council house/flat	120 (49)	105 (47)	15 (65)		30 (56)	90 (47)	
Other	37 (15)	34 (15)	3 (13)		7 (13)	30 (16)	
**Occupation, *n* (%)**							
Education	189 (77)	171 (76)	18 (78)	1.000	39 (72)	150 (78)	0.013
Employment	40 (16)	36 (16)	4 (17)		6 (11)	34 (18)	
Not in education/employment	18 (7)	17 (8)	1 (4)		9 (17)	9 (5)	
**Parental vital status, *n* (%)** *n* = 230^a^ (vs Both parents alive)							
Death of one/both parents	95 (41)	93 (45)	2 (10)	0.002	20 (40)	75 (42)	0.832
**Age at ART initiation (years), Median [IQR]**	7.5 [3.2, 11.5]	7.5 [3.3, 11.7]	5.4 [0.8, 11.5]	0.127	7.6 [2.7, 12.2]	7.4 [3.3, 11.4]	0.819
**Years since ART initiation, Median [IQR]**	11.3 [6.9, 15.8]	11.0 [6.8, 15.5]	13.7 [8.0, 17.9]	0.201	11.5 [7.0, 17.0]	11.0 [6.0, 15.6]	0.600
**Total number of tablets taken per day, *n* (%)** (vs 1 tablet)							
≥2	198 (80)	180 (80)	18 (78)	0.786	44 (81)	154 (80)	0.783
**Type of regimen, *n* (%)**							
NNRTI-based regimen	70 (28)	64 (29)	6 (26)	0.981	8 (15)	62 (32)	0.018
PI-based regimen	136 (55)	122 (54)	14 (61)		31 (57)	105 (54)	
INSTI-based regimen	17 (7)	16 (7)	1 (4)		7 (13)	10 (5)	
Other	24 (10)	22 (10)	2 (9)		8 (15)	16 (8)	
**Viral load, *n* (%)** *n* = 233^a^ (vs ≥50 copies/ml) <50 copies/ml	163 (70)	149 (70)	14 (67)	0.730	28 (53)	135 (75)	0.002
**CD4 cell count (cells/mm^3^),** **Median [IQR]** *n* = 213^a^	598 [439, 785]	609 [439, 783]	564 [468, 924]	0.611	500 [363, 683]	636 [472, 849]	0.008
**CDC Stage at time of interview, *n* (%)** (vs Stage N/A/B)							
Stage C	65 (26)	55 (25)	10 (43)	0.050	19 (35)	46 (24)	0.094
**Type of care at time of interview, *n* (%)** (vs Paediatric)							
Adolescent/Adult	132 (53)	122 (54)	10 (43)	0.314	30 (56)	102 (53)	0.725
**EQ5D-5L Health-related Quality of Life Scores, Median [IQR]** *n* = 240^a^							
EQ5D Index Score	0.94 [0.85, 1.00]	0.94 [0.85, 1.00]	0.92 [0.90, 1.00]	0.485	0.87 [0.73, 1.00]	0.94 [0.86, 1.00]	<0.001
EQ-VAS Score	61 [80, 90]	80 [65, 90]	74 [50, 85]	0.091	69 [50, 80]	80 [69, 90]	<0.001
**ACS-2 Proportions, Median [IQR]** *n* = 239^a^							
Productive coping usage	66 [58, 74]	66 [59, 74]	60 [54, 68]	0.010	64 [58, 72]	66 [58, 74]	0.435
Productive coping helpfulness	66 [58, 76]	68 [58, 76]	62 [50, 72]	0.059	62 [58, 70]	68 [58, 76]	0.029
Non-productive coping usage	55 [45, 65]	55 [45, 65]	50 [43, 60]	0.120	60 [48, 70]	53 [45, 65]	0.009
Non-productive coping helpfulness	40 [35, 48]	40 [34, 48]	38 [35, 48]	0.775	43 [38, 55]	40 [33, 48]	0.022
**Rosenberg Self-esteem Score, Median [IQR]** *n* = 240^a^	19 [16, 23]	19 [16, 23]	20 [18, 23]	0.484	18 [15, 22]	20 [16, 23]	0.011
**Last month adherent, *n* (%)^c^**	158 (64)	144 (64)	14 (61)	0.745	19 (35)	139 (72)	<0.001
**3-day adherent, *n* (%)^d^**	172 (70)	157 (70)	15 (65)	0.628	32 (59)	140 (73)	0.061
**BMQ-HAART, median [IQR]**							
Mean Necessity item score	3.9 [3.5, 4.4]	3.9 [3.6, 4.4]	2.8 [2.6, 2.9]	<0.001	3.8 [3.4, 4.1]	3.9 [3.5, 4.4]	0.117
Mean Concerns item score	2.5 [2.1, 3.0]	2.4 [2.0, 2.9]	2.9 [2.5, 3.4]	0.002	3.4 [3.2, 3.5]	2.3 [1.9, 2.5]	<0.001
Necessity-Concerns differential	1.4 [0.7, 2.0]	1.5 [1.0, 2.1]	−0.2 [−0.7, 0.4]	<0.001	0.4 [−0.1, 0.8]	1.6 [1.2, 2.1]	<0.001

Note: ACS-2, Adolescent Coping Scale Second Edition; ART, antiretroviral therapy; BMQ-HAART, Beliefs About Medicine Questionnaire – Highly Active Antiretroviral Therapy version; CDC, Centers for Disease Control and Prevention; EQ5D-5L – EuroQol 5-Dimension 5-level version; EQ-VAS, EuroQol – visual analogue scale; NNRTI, non-nucleoside reverse transcriptase inhibitor; PI, protease inhibitor; INSTI, integrase inhibitor; SD, standard deviation; UK, United Kingdom.

a Sample size is less than the total due to missing data.

b Two-sided *p*-value for *χ*^2^ or Fisher’s exact test for categorical variables, and Wilcoxon rank sum test for continuous variables, comparing high and low total Necessity score or high and low total Concerns score groups.

c Did not miss more than two doses of ART in a row month prior to the interview.

d Did not miss any doses of ART in the three days prior to the interview.

**Table 2:** Odds ratios of being Last Month Adherent (did not miss more than two doses of ART in a row in the month prior to interview) and having a viral load (VL) < 50 copies/ml for participants with BMQ-HAART high Necessity and high Concerns scores in univariable models [(Model 1), adjusted for high Concerns/high Necessity score respectively (Model 2) and adjusted for all sociodemographic, clinical and psychosocial variables (Model 3)].

	Last Month Adherent^d^				VL < 50 copies/ml^e^		
	Odds ratio	95% confidence interval	*p*-value		Odds ratio	95% confidence interval	*p*-value
BMQ-HAART High Necessity Score^a^ (vs. Low Necessity Score)							
Model 1 (Univariable)	1.16	0.48–2.79	0.745		1.18	0.46–3.07	0.730
Model 2 (Adjusted for High Concerns Score)	0.84	0.32–2.18	0.718		0.94	0.35–2.53	0.902
Model 3 (Fully adjusted)^c^	1.34	0.34–5.28	0.679		1.42	0.36–5.67	0.615
BMQ-HAART High Concerns Score^b^ (vs. Low Concerns Score)							
Model 1 (Univariable)	0.21	0.11–0.40	<0.001		0.37	0.20–0.71	0.002
Model 2 (Adjusted for High Necessity Score)	0.21	0.11–0.40	<0.001		0.37	0.19–0.71	0.003
Model 3 (Fully adjusted)^c^	0.19	0.07–0.47	<0.001		0.61	0.25–1.52	0.293

Note: BMQ-HAART, Beliefs About Medicine Questionnaire – Highly Active Antiretroviral Therapy version.

a High Necessity Score = BMQ-HAART total Necessity score > 24, Low Necessity Score = BMQ-HAART total Necessity score ≤ 24.

b High Concerns Score = BMQ-HAART total Concerns score >33, Low Concerns Score = BMQ-HAART total Concerns score ≤ 33.

c Age at ART initiation, type of ART regimen, occupation and type of care were excluded from the multivariable model due to collinearity concerns.

d 247 participants included in Model 1 and Model 2, 187 participants in Model 3.

e 233 participants were included in Model 1 and Model 2, 186 participants included in Model 3.

## Data Availability

The AALPHI data are held at MRC CTU at UCL, which encourages optimal use of data by employing a controlled access approach to data sharing, incorporating a transparent and robust system to review requests and provide secure data access consistent with the relevant ethics committee approvals. The rationale for this approach has been published (doi:10.1186/s13063-015-0604-6). Ethics committee approval for use of AALPHI data restrict the ability for AALPHI data to be shared publicly without request. Rather, ethics approval does allow a controlled access approach. All requests for data are considered and can be initiated by contacting mrcctu.datareleaserequest@ucl.ac.uk.

## Sustainable development goals

SDG 3: Good health and well-being
