# Supplementary material for: Beliefs about antiretroviral therapy and their association with adherence in young people living with perinatal HIV in England: a cross-sectional analysis
Source: AIDS Care. Author manuscript; Available in PMC 2024 Dec 9. (PMC7617179; doi:10.1080/09540121.2023.2300984)
Supplement: Appendices [file EMS198641-supplement-Appendices.pdf]

# Appendices

## *Appendix A. Properties of instruments/scales used to measure psychosocial and quality of life variables.*

| Variable | Instrument(s)/Scales | Statements/Questions | Scoring | Theoretical Range and Interpretation |
|---|---|---|---|---|
| Beliefs about ART | BMQ-HAART Necessity Scale (Horne et al., 2004) | 8 statements e.g., "These medicines keep me alive" | 5-point Likert-type scale, 1 = Strongly Disagree, 5 = Strongly Agree. Scores totalled. | Higher total score indicates stronger beliefs in the necessity of ART. Theoretical range: 8–40. |
| | BMQ-HAART Concerns Scale (Horne et al., 2004) | 11 statements e.g., "Using these medicines is embarrassing" | 5-point Likert-type scale, 1 = Strongly Disagree, 5 = Strongly Agree. Scores totalled. | Higher total score indicates stronger concerns regarding ART. Theoretical range: 11–55. |
| Health-related QoL | EuroQol 5-Dimension 5-level version (EQ5D-5L) Descriptive System (Herdman et al., 2011) | 5 dimensions (Mobility, Self-care, Activities, Pain/Discomfort, Anxiety/Depression) | 5-point scale 1 = No problems, 5 = Extreme Problems 5 scores for the dimensions converted to EQ5D Index Score based on UK value set (Devlin et al., 2018; EuroQol, 2020). | Higher index score indicates better health-related QoL. Negative index score represents a health profile perceived to be worse than death. UK value set EQ5D index score theoretical range: −0.285 (for score of 5 on all dimensions) – 1.000 (for score of 1 on all dimensions i.e., perfect health) |
| | EuroQol-VAS (EQ-VAS) (Herdman et al., 2011) | Participants asked to mark how good or bad their health was on day of interview. | Vertical VAS, 0 ("The worst imaginable health state")−100 ("The best imaginable health state"). | Higher score represents better self-reported QoL. Theoretical range: 0–100. |
| Coping | ACS-2 Short Form – Productive Coping Usage Scale (Frydenberg & Lewis, 2011a) | How often participants use each of 10 productive coping strategies when dealing with concerns or problems (e.g., "Ask a teacher or other professional for help") | 5-point scale, 1 = Never, 5 = Very often. Scores for 10 items totalled, divided by 10 and multiplied by 20 to obtain % score(Frydenberg & Lewis, 2011b). | Higher % score = greater usage of productive coping strategies. Theoretical range: 20% (Never)−100% (Very Often) |
| | ACS-2 Short Form – Productive Coping Helpfulness Scale (Frydenberg & Lewis, 2011a) | How often participants find the 10 productive coping strategies (as above) helpful. | As above. | Higher % score = greater helpfulness of productive coping strategies. Theoretical range: 20% (Never) – 100% (Very often) |
| | ACS-2 Short Form – Non-productive Coping Usage Scale (Frydenberg & Lewis, 2011a) | Participants asked to rate how often they use each of 8 non-productive coping strategies when dealing with concerns or problems (e.g., "Blame myself"). | 5-point scale, 1 = Never, 5 = Very often. Scores for 8 items totalled, divided by 8 and multiplied by 20 to obtain % score.(Frydenberg & Lewis, 2011b) | Higher % score = greater usage of non-productive coping strategies. Theoretical range: 20% (Never)-100% (Very Often) |
| | ACS-2 Short Form – Non-productive Coping Helpfulness Scale (Frydenberg & Lewis, 2011a) | Participants asked to rate how often they find the 8 non-productive coping strategies (as above) helpful. | As above. | Higher % score = greater helpfulness of non-productive coping strategies. Theoretical range: 20% (Never)−100% (Very Often) |
| Self-Esteem | Rosenberg Self-Esteem Scale (Rosenberg, 1965) | Participants asked to rate the extent to which they agree with each of 10 statements e.g., "I feel that I have a number of good qualities" | 4-point scale, 0 = Strongly Disagree-3 = Strongly Agree. Scores summed to obtain total self-esteem score. | Higher score indicates higher self-esteem. Theoretical range: 0–30. |

Note: ART, antiretroviral therapy, BMQ-HAART, Beliefs About Medicine Questionnaire – Highly Active Antiretroviral Therapy version, QoL, Quality of Life, ACS-2, Adolescent Coping Scale – 2nd Edition; EQ5D-5L – EuroQol 5-Dimension 5-level version; EQ-VAS, EuroQol-visual analogue scale.

## Appendix B. Unadjusted odds ratios of being Last Month Adherent (did not miss more than two doses of ART in a row in month prior to interview) and having a viral load <50 copies/ml (within 6 months before or after the interview date), for sociodemographic, clinical and psychosocial variables (Model 1).

| Variable | Last Month Adherent[b] Crude Odds ratio (OR) | Last Month Adherent[b] 95% confidence interval (CI) | Last Month Adherent[b] *p*-Value | Viral load <50 copies/ml[c] Crude Odds ratio (OR) | Viral load <50 copies/ml[c] 95% confidence interval (CI) | Viral load <50 copies/ml[c] *p*-Value |
|---|---|---|---|---|---|---|
| **Sex** (vs Male) | | | | | | |
| Female | 0.72 | 0.42–1.24 | 0.237 | 0.81 | 0.45–1.43 | 0.460 |
| **Age at interview (per year increase)** | 0.86 | 0.77–0.95 | 0.004 | 0.92 | 0.82–1.02 | 0.115 |
| **Ethnicity** (vs Non-Black) | | | | | | |
| Black | 0.58 | 0.25–1.36 | 0.209 | 0.87 | 0.37–2.08 | 0.758 |
| **Birthplace** (vs Born in UK) | | | | | | |
| Born Outside the UK | 0.88 | 0.52–1.49 | 0.623 | 1.18 | 0.67–2.07 | 0.572 |
| **Parental vital status** (vs Both parents alive) | | | | | | |
| One/both parents died | 0.87 | 0.50–1.51 | 0.614 | 1.25 | 0.68–2.29 | 0.469 |
| **Living Situation** (vs Other)[a] | | | | | | |
| Housing association/council house/flat | 1.09 | 0.65–1.84 | 0.743 | 1.24 | 0.71–2.18 | 0.449 |
| **Occupation** (vs Other)[a] | | | | | | |
| Education | 1.35 | 0.74–2.46 | 0.333 | 1.63 | 0.86–3.07 | 0.134 |
| **Total number of tablets taken per day** (vs 1 tablet) | | | | | | |
| ≥2 tablets | 0.45 | 0.21–0.92 | 0.030 | 0.47 | 0.21–1.03 | 0.059 |
| **Type of ART regimen** (vs Other)[a] | | | | | | |
| PI-based regimen | 0.33 | 0.19–0.57 | <0.001 | 0.37 | 0.21–0.68 | 0.001 |
| **CDC Stage at time of interview** (vs Stage N/A/B) | | | | | | |
| Stage C | 1.04 | 0.58–1.88 | 0.899 | 0.66 | 0.36–1.23 | 0.191 |
| **Type of care at time of interview** (vs Paediatric) | | | | | | |
| Adolescent/Adult | 0.55 | 0.32–0.93 | 0.026 | 0.60 | 0.34–1.05 | 0.075 |
| **Years since ART initiation** (per year increase) | 0.96 | 0.91–1.01 | 0.079 | 0.96 | 0.91–1.02 | 0.172 |
| **Age at ART initiation (per year increase)** | 1.01 | 0.96–1.06 | 0.734 | 1.02 | 0.96–1.07 | 0.534 |
| **CD4 Count (per 50 cells/mm$^3$ increase)** | 1.09 | 1.04–1.15 | 0.001 | 1.18 | 1.10–1.26 | <0.001 |
| **EQ5D-5L Health-related Quality of Life Score** | | | | | | |
| EQ5D Index Score (per 0.1 increase) | 1.25 | 1.03–1.52 | 0.022 | 1.38 | 1.12–1.70 | 0.002 |
| EQ-VAS (per 1% increase) | 1.03 | 1.02–1.05 | <0.001 | 1.03 | 1.01–1.04 | <0.001 |
| **ACS-2 Score** | | | | | | |
| Productive Coping Usage (per 1% increase) | 1.01 | 0.99–1.04 | 0.328 | 1.01 | 0.99–1.04 | 0.369 |
| Productive Coping Helpfulness (per 1% increase) | 1.03 | 1.01–1.05 | 0.011 | 1.01 | 0.99–1.03 | 0.341 |
| Non-productive Coping Usage (per 1% increase) | 0.97 | 0.95–0.98 | <0.001 | 0.97 | 0.95–0.99 | 0.006 |
| Non-productive Coping Helpfulness (per 1% increase) | 0.99 | 0.97–1.01 | 0.315 | 0.98 | 0.96–1.00 | 0.058 |
| **Rosenberg Self-Esteem Score (per 5-point increase)** | 1.30 | 1.01–1.68 | 0.044 | 1.60 | 1.20–2.15 | 0.002 |

Note: ACS-2, Adolescent Coping Scale Second Edition; ART, antiretroviral therapy; CDC, Centers for Disease Control and Prevention; EQ5D-5L – EuroQol 5-Dimension 5-level version; EQ-VAS, EuroQol – visual analogue scale; NNRTI, non-nucleoside reverse transcriptase inhibitor; PI, protease inhibitor; UK, United Kingdom.

[a]Collapsed into a binary variable due to small numbers in some categories.

[b]247 participants in all models apart from parental vital status ($n = 230$), CD4 cell count ($n = 213$), EQ5D-5L Health-related Quality of Life Scores ($n = 240$), ACS-2 Scores ($n = 239$) and Rosenberg Self-Esteem Score ($n = 240$).

[c]233 participants in all models apart from parental vital status ($n = 216$), CD4 cell count ($n = 212$), EQ5D-5L Health-related Quality of Life Scores ($n = 226$), ACS-2 Scores ($n = 225$) and Rosenberg Self-Esteem Score ($n = 226$).

***Appendix C. Adjusted odds ratios of being Last Month Adherent (did not miss greater than 2 doses in month prior to interview) for BMQ-HAART Necessity and Concerns scores, adjusted for individual sociodemographic, clinical, and psychosocial variables (Model 2).***

| | Sociodemographic/clinical/psychosocial variable | | | High Necessity Score[a] (vs Low Necessity Score) | | | High Concerns Score[b] (vs Low Concerns Score) | | |
|---|---|---|---|---|---|---|---|---|---|
| Sociodemographic/ Clinical/Psychosocial Variables | Adjusted odds ratio of being Last Month Adherent | 95% CI for adjusted odds ratio of being Last Month Adherent | *p*-value | Adjusted odds ratio of being Last Month Adherent | 95% CI for adjusted odds ratio of being Last Month Adherent | *p*-value | Adjusted odds ratio of being Last Month Adherent | 95% CI for adjusted odds ratio of being Last Month Adherent | *p*-value |
| **None (both BMQ-HAART variables only)** | – | – | – | 0.84 | 0.32–2.18 | 0.718 | 0.21 | 0.11–0.40 | <0.001 |
| **Sex** (vs Male) | | | | | | | | | |
| Female | 0.72 | 0.41–1.27 | 0.258 | 0.84 | 0.32–2.21 | 0.726 | 0.21 | 0.11–0.40 | <0.001 |
| **Age at interview (per year increase)** | 0.87 | 0.78–0.97 | 0.010 | 0.89 | 0.34–2.32 | 0.817 | 0.22 | 0.11–0.42 | <0.001 |
| **Ethnicity** (vs Non-Black) | | | | | | | | | |
| Black | 0.66 | 0.27–1.60 | 0.359 | 0.83 | 0.32–2.16 | 0.704 | 0.21 | 0.11–0.41 | <0.001 |
| **Birthplace** (vs Born in UK) | | | | | | | | | |
| Born Outside the UK | 0.79 | 0.45–1.38 | 0.409 | 0.95 | 0.33–2.21 | 0.736 | 0.20 | 0.11–0.39 | <0.001 |
| **Living Situation** (vs Other)[c] | | | | | | | | | |
| Housing association/ council house/flat | 1.22 | 0.70–2.13 | 0.481 | 0.87 | 0.33–2.27 | 0.771 | 0.20 | 0.11–0.39 | <0.001 |
| **Occupation** (vs Other) | | | | | | | | | |
| Education[c] | 1.27 | 0.37–2.40 | 0.464 | 0.84 | 0.32–2.20 | 0.730 | 0.21 | 0.11–0.40 | <0.001 |
| **Parental Vital Status** (vs Both parents alive) | | | | | | | | | |
| One/both parents died | 0.83 | 0.45–1.51 | 0.534 | 1.04 | 0.37–2.92 | 0.944 | 0.17 | 0.09–0.34 | <0.001 |
| **Total number of tablets taken per day** (vs 1 tablet) | | | | | | | | | |
| ≥2 tablets | 0.42 | 0.20–0.91 | 0.028 | 0.85 | 0.33–2.25 | 0.750 | 0.20 | 0.10–0.39 | <0.001 |
| **Type of ART regimen** (vs Other)[c] | | | | | | | | | |
| PI-based regimen | 0.30 | 0.16–0.54 | <0.001 | 0.79 | 0.30–2.08 | 0.638 | 0.19 | 0.09–0.37 | <0.001 |
| **CDC Stage** (vs Stage N/ A/B) | | | | | | | | | |
| Stage C | 1.24 | 0.65–2.35 | 0.512 | 0.87 | 0.33–2.28 | 0.781 | 0.20 | 0.11–0.39 | <0.001 |
| **Type of care at time of interview** (vs Paediatric) | | | | | | | | | |
| Adolescent/Adult | 0.53 | 0.30–0.93 | 0.027 | 0.91 | 0.35–2.36 | 0.852 | 0.20 | 0.11–0.40 | <0.001 |
| **Years since ART initiation (per year increase)** | 0.95 | 0.91–1.01 | 0.086 | 0.79 | 0.30–2.05 | 0.626 | 0.21 | 0.11–0.39 | <0.001 |
| **Age at ART initiation (per year increase)** | 1.01 | 0.96–1.07 | 0.643 | 0.82 | 0.31–2.14 | 0.686 | 0.21 | 0.11–0.39 | <0.001 |
| **CD4 Count (per 50 cells/mm$^3$ increase)** | 1.08 | 1.02–1.14 | 0.011 | 1.17 | 0.40–3.38 | 0.776 | 0.19 | 0.09–0.39 | <0.001 |
| **EQ5D-5L Health-related Quality of Life Scores** | | | | | | | | | |
| EQ5D Index Score (per 0.1 increase) | 1.13 | 0.92–1.39 | 0.227 | 0.90 | 0.33–2.45 | 0.837 | 0.22 | 0.11–0.43 | <0.001 |
| EQ-VAS (per 1% increase) | 1.03 | 1.01–1.04 | 0.001 | 0.71 | 0.25–2.00 | 0.518 | 0.24 | 0.12–0.48 | <0.001 |
| **ACS-2 Score** | | | | | | | | | |
| Productive Coping Usage (per 1% increase) | 1.01 | 0.99–1.04 | 0.356 | 0.80 | 0.30–2.12 | 0.655 | 0.21 | 0.11–0.40 | <0.001 |
| Productive Coping Helpfulness (per 1% increase) | 1.03 | 1.00–1.05 | 0.033 | 0.75 | 0.29–1.97 | 0.560 | 0.22 | 0.11–0.42 | <0.001 |
| Non-productive Coping Usage (per 1% increase) | 0.97 | 0.95–0.99 | 0.004 | 1.05 | 0.39–2.84 | 0.928 | 0.24 | 0.12–0.47 | <0.001 |

(*Continued*)

Continued.

| Sociodemographic/ Clinical/Psychosocial Variables | Sociodemographic/clinical/psychosocial variable | | | High Necessity Score[a] (vs Low Necessity Score) | | | High Concerns Score[b] (vs Low Concerns Score) | | |
|---|---|---|---|---|---|---|---|---|---|
| | Adjusted odds ratio of being Last Month Adherent | 95% CI for adjusted odds ratio of being Last Month Adherent | p-value | Adjusted odds ratio of being Last Month Adherent | 95% CI for adjusted odds ratio of being Last Month Adherent | p-value | Adjusted odds ratio of being Last Month Adherent | 95% CI for adjusted odds ratio of being Last Month Adherent | p-value |
| Non-productive Coping Helpfulness (per 1% increase) | 1.00 | 0.98–1.02 | 0.955 | 0.87 | 0.33–2.26 | 0.769 | 0.21 | 0.11–0.41 | <0.001 |
| **Rosenberg Self-Esteem Score (per 5-point increase)** | 1.20 | 0.91–1.57 | 0.191 | 0.86 | 0.31–2.35 | 0.767 | 0.21 | 0.11–0.40 | <0.001 |

Note: ACS-2, Adolescent Coping Scale Second Edition; ART, antiretroviral therapy; BMQ-HAART, Beliefs About Medicine Questionnaire – Highly Active Antiretroviral Therapy version; CDC, Centers for Disease Control and Prevention; EQ5D-5L – EuroQol 5-Dimension 5-level version; EQ-VAS, EuroQol-visual analogue scale; NNRTI, non-nucleoside reverse transcriptase inhibitor; PI, protease inhibitor; UK, United Kingdom, CI, confidence interval.
[a]High Necessity score = BMQ-HAART total Necessity score >24, Low Necessity score = BMQ-HAART total Necessity score ≤24.
[b]High Concerns score = BMQ-HAART total Concerns score >33, Low Concerns score = BMQ-HAART total Concerns score ≤33.
[c]Collapsed into a binary variable due to small numbers in some categories.
A total of 247 participants in all models apart from parental vital status (*n* = 230), CD4 cell count (*n* = 213), EQ5D-5L Health-related Quality of Life Scores (*n* = 240), ACS-2 Scores (*n* = 239) and Rosenberg Self-Esteem Score (*n* = 240).

## *Appendix D. Adjusted odds ratios of having a viral load <50 copies/ml (within 6 months before or after the interview date) for BMQ-HAART Necessity and Concerns scores, adjusted for individual sociodemographic, clinical, and psychosocial variables (Model 2).*

| Sociodemographic /Clinical/Psychosocial Variables | Sociodemographic/clinical/psychosocial variable | | | High Necessity Score[a] (vs Low Necessity Score) | | | High Concerns Score[b] (vs Low Concerns Score) | | |
|---|---|---|---|---|---|---|---|---|---|
| | Adjusted odds ratio of VL <50 copies/ml | 95% CI for adjusted odds ratio of VL <50 copies/ml | p-value | Adjusted odds ratio of VL < 50 copies/ml | 95% CI for adjusted odds ratio of VL <50 copies/ml | p-value | Adjusted odds ratio of VL <50 copies/ml | 95% CI for adjusted odds ratio of VL <50 copies ml | p-value |
| **None** (both BMQ-HAART variables only) | – | – | – | 0.94 | 0.35–2.53 | 0.902 | 0.37 | 0.19–0.71 | 0.003 |
| **Sex** (vs Male) | | | | | | | | | |
| Female | 0.82 | 0.46–1.48 | 0.514 | 0.93 | 0.34–2.52 | 0.889 | 0.37 | 0.20–0.71 | 0.003 |
| **Age at interview (per year increase)** | 0.93 | 0.83–1.04 | 0.189 | 0.99 | 0.37–2.68 | 0.986 | 0.39 | 0.20–0.74 | 0.004 |
| **Ethnicity** (vs Non-Black) | | | | | | | | | |
| Black | 0.97 | 0.40–2.35 | 0.950 | 0.94 | 0.35–2.53 | 0.901 | 0.37 | 0.19–0.71 | 0.003 |
| **Birthplace** (vs Born in UK) | | | | | | | | | |
| Born Outside the UK | 1.12 | 0.63–1.99 | 0.710 | 0.93 | 0.34–2.51 | 0.887 | 0.37 | 0.20–0.71 | 0.003 |
| **Living Situation** (vs Other)[c] | | | | | | | | | |
| Housing association/ council house/flat | 1.35 | 0.75–2.41 | 0.320 | 1.01 | 0.37–2.75 | 0.990 | 0.36 | 0.19–0.69 | 0.002 |
| **Occupation** (vs Other)[c] | | | | | | | | | |
| Education | 1.56 | 0.81–2.99 | 0.182 | 0.97 | 0.36–2.62 | 0.949 | 0.38 | 0.20–0.72 | 0.003 |
| **Parental Vital Status** (vs Both parents alive) | | | | | | | | | |
| One/both parents died | 1.22 | 0.65–2.31 | 0.536 | 1.16 | 0.41–3.30 | 0.782 | 0.32 | 0.16–0.63 | 0.001 |
| **Total number of tablets taken per day** (vs 1 tablet) | | | | | | | | | |
| ≥2 tablets | 0.47 | 0.21–1.04 | 0.063 | 0.97 | 0.35–2.63 | 0.946 | 0.37 | 0.19–0.71 | 0.003 |
| **Type of ART regimen** (vs Other)[c] | | | | | | | | | |
| PI-based regimen | 0.38 | 0.21–0.69 | 0.002 | 0.94 | 0.34–2.55 | 0.900 | 0.38 | 0.19–0.73 | 0.004 |
| **CDC Stage** (vs Stage N/A/B) | | | | | | | | | |
| Stage C | 0.70 | 0.37–1.32 | 0.276 | 0.88 | 0.32–2.41 | 0.802 | 0.38 | 0.20–0.72 | 0.003 |
| **Type of care at time of interview** (vs Paediatric) | | | | | | | | | |
| Adolescent/Adult | 0.60 | 0.34–1.09 | 0.091 | 1.03 | 0.38–2.79 | 0.952 | 0.38 | 0.20–0.72 | 0.003 |

*(Continued)*

Continued.

| Sociodemographic /Clinical/Psychosocial Variables | Sociodemographic/clinical/psychosocial variable | | | High Necessity Score[a] (vs Low Necessity Score) | | | High Concerns Score[b] (vs Low Concerns Score) | | |
|---|---|---|---|---|---|---|---|---|---|
| | Adjusted odds ratio of VL <50 copies/ml | 95% CI for adjusted odds ratio of VL <50 copies/ml | *p*-value | Adjusted odds ratio of VL < 50 copies/ml | 95% CI for adjusted odds ratio of VL <50 copies/ml | *p*-value | Adjusted odds ratio of VL <50 copies/ml | 95% CI for adjusted odds ratio of VL <50 copies ml | *p*-value |
| **Years since ART initiation (per year increase)** | 0.96 | 0.91–1.02 | 0.192 | 0.90 | 0.33–2.44 | 0.840 | 0.37 | 0.19–0.71 | 0.003 |
| **Age at ART initiation (per year increase)** | 1.02 | 0.96–1.08 | 0.482 | 0.91 | 0.33–2.46 | 0.848 | 0.37 | 0.19–0.70 | 0.002 |
| **CD4 Count (per 50 cells/mm$^3$ increase)** | 1.17 | 1.09–1.25 | <0.001 | 1.06 | 0.35–3.20 | 0.921 | 0.42 | 0.21–0.88 | 0.021 |
| **EQ5D-5L Health-related Quality of Life Scores** | | | | | | | | | |
| EQ5D Index Score (per 0.1 increase) | 1.30 | 1.05–1.60 | 0.015 | 1.29 | 0.46–3.59 | 0.628 | 0.41 | 0.21–0.81 | 0.010 |
| EQ-VAS (per 1% increase) | 1.02 | 1.01–1.04 | 0.003 | 0.97 | 0.34–2.76 | 0.947 | 0.42 | 0.21–0.82 | 0.011 |
| **ACS-2 Score** | | | | | | | | | |
| Productive Coping Usage (per 1% increase) | 1.01 | 0.99–1.04 | 0.389 | 0.88 | 0.32–2.41 | 0.807 | 0.40 | 0.21–0.76 | 0.005 |
| Productive Coping Helpfulness (per 1% increase) | 1.01 | 0.98–1.03 | 0.553 | 0.91 | 0.34–2.48 | 0.859 | 0.41 | 0.21–0.78 | 0.007 |
| Non-productive Coping Usage (per 1% increase) | 0.98 | 0.96–1.00 | 0.025 | 1.10 | 0.40–3.03 | 0.859 | 0.47 | 0.24–0.92 | 0.027 |
| Non-productive Coping Helpfulness (per 1% increase) | 0.98 | 0.96–1.01 | 0.180 | 0.97 | 0.35–2.63 | 0.946 | 0.43 | 0.22–0.85 | 0.015 |
| **Rosenberg Self-Esteem Score (per 5-point increase)** | 1.52 | 1.13–2.05 | 0.006 | 1.23 | 0.44–3.46 | 0.692 | 0.38 | 0.20–0.75 | 0.005 |

Note: ACS-2, Adolescent Coping Scale Second Edition; ART, antiretroviral therapy; BMQ-HAART, Beliefs About Medicine Questionnaire – Highly Active Antiretroviral Therapy version; CDC, Centers for Disease Control and Prevention; EQ5D-5L, EuroQol 5-Dimension 5-level version; EQ-VAS, EuroQol-visual analogue scale; NNRTI, non-nucleoside reverse transcriptase inhibitor; PI, protease inhibitor; UK, United Kingdom, CI, confidence interval; VL, viral load.

[a]High Necessity score = BMQ-HAART total Necessity score >24, Low Necessity score = BMQ-HAART total Necessity score ≤24.

[b]High Concerns score = BMQ-HAART total Concerns score >33, Low Concerns score = BMQ-HAART total Concerns score ≤33.

[c]Collapsed into a binary variable due to small numbers in some categories.

A total of 233 participants in all models apart from parental vital status ($n = 216$), CD4 cell count ($n = 212$), EQ5D-5L Health-related Quality of Life Scores ($n = 226$), ACS-2 Scores ($n = 225$) and Rosenberg Self-Esteem Score ($n = 226$).

## Appendix E. Adjusted (Model 3) odds ratios of being Last Month Adherent (did not miss more than two doses of ART in a row in month prior to interview) and adjusted odds ratios of having a viral load <50 copies/ml (within 6 months before or after the interview date), for BMQ-HAART Necessity and Concerns scores and all sociodemographic, clinical and psychosocial variables included in a multivariable model.

| | Last Month Adherent ($n = 187$) | | | VL <50 copies/ml ($n = 186$) | | |
|---|---|---|---|---|---|---|
| Variable | Adjusted Odds ratio | 95% Confidence interval | *p*-Value | Adjusted Odds ratio | 95% Confidence interval | *p*-value |
| **BMQ-HAART** (vs Low score) | | | | | | |
| High Necessity Score[a] | 1.34 | 0.34–5.28 | 0.679 | 1.42 | 0.36–5.67 | 0.615 |
| High Concerns Score[b] | 0.19 | 0.07–0.47 | <0.001 | 0.61 | 0.25–1.52 | 0.293 |
| **Sex** (vs Male) | | | | | | |
| Female | 0.92 | 0.42–2.05 | 0.845 | 1.32 | 0.59–2.98 | 0.500 |
| **Age at interview** (per year increase) | 0.94 | 0.81–1.11 | 0.471 | 0.99 | 0.84–1.16 | 0.888 |
| **Ethnicity** (vs Non-Black) | | | | | | |
| Black | 0.81 | 0.25–2.62 | 0.721 | 0.60 | 0.18–1.96 | 0.400 |
| **Birthplace** (vs Born in UK) | | | | | | |
| Born Outside the UK | 0.61 | 0.24–1.54 | 0.295 | 1.31 | 0.53–3.26 | 0.562 |
| **Parental vital status** (vs Both parents alive) | | | | | | |
| One/both parents died | 1.10 | 0.50–2.45 | 0.812 | 1.59 | 0.70–3.59 | 0.270 |
| **Living Situation** (vs Other)[d] | | | | | | |
| Housing association/council house/flat | 1.23 | 0.54–2.78 | 0.616 | 1.68 | 0.73–3.84 | 0.221 |
| **Occupation** (vs Other)[c,d] | | | | | | |
| Education | – | – | – | – | – | – |
| **Total number of tablets taken per day** (vs 1 tablet) | | | | | | |
| $\geq$2 tablets | 0.38 | 0.13–1.11 | 0.077 | 0.33 | 0.11–1.00 | 0.050 |
| **Type of ART regimen** (vs Other)[c,d] | | | | | | |
| PI-based regimen | – | – | – | – | – | – |
| **CDC Stage at time of interview** (vs Stage N/A/B) | | | | | | |
| Stage C | 1.23 | 0.48–3.11 | 0.668 | 0.60 | 0.24–1.51 | 0.279 |
| **Type of care at time of interview** (vs Paediatric)[c] | | | | | | |
| Adolescent/Adult | – | – | – | – | – | – |
| **Years since ART initiation (per year increase)** | 0.97 | 0.88–1.06 | 0.481 | 0.99 | 0.91–1.09 | 0.913 |
| **Age at ART initiation (per year increase)**[c] | – | – | – | – | – | – |
| **CD4 Count (per 50 cells/mm$^3$ increase)** | 1.06 | 0.99–1.14 | 0.082 | 1.16 | 1.07–1.25 | <0.001 |
| **EQ5D-5L Health-related Quality of Life Score** | | | | | | |
| EQ5D Index Score (per 0.1 increase) | 1.00 | 0.75–1.36 | 0.973 | 1.06 | 0.79–1.42 | 0.692 |
| EQ-VAS (per 1% increase) | 1.02 | 1.00–1.04 | 0.121 | 1.02 | 1.00–1.04 | 0.093 |
| **ACS-2 Score** | | | | | | |
| Productive Coping Usage (per 1% increase) | 0.99 | 0.94–1.04 | 0.569 | 0.99 | 0.94–1.05 | 0.820 |
| Productive Coping Helpfulness (per 1% increase) | 1.02 | 0.98–1.07 | 0.343 | 1.01 | 0.96–1.06 | 0.810 |
| Non-productive Coping Usage (per 1% increase) | 0.96 | 0.93–1.00 | 0.049 | 1.00 | 0.97–1.04 | 0.837 |
| Non-productive Coping Helpfulness (per 1% increase) | 1.03 | 0.99–1.07 | 0.192 | 0.99 | 0.96–1.03 | 0.614 |
| **Rosenberg Self-Esteem Score (per 5-point increase)** | 0.68 | 0.41–1.13 | 0.135 | 1.40 | 0.83–2.38 | 0.208 |

Note: ACS-2, Adolescent Coping Scale Second Edition; ART, antiretroviral therapy; BMQ-HAART, Beliefs About Medicine Questionnaire – Highly Active Antiretroviral Therapy version; CDC, Centers for Disease Control and Prevention; EQ5D-5L, EuroQol 5-Dimension 5-level version; EQ-VAS, EuroQol-visual analogue scale; NNRTI, non-nucleoside reverse transcriptase inhibitor; PI, protease inhibitor; UK, United Kingdom, VL, viral load.
[a]High Necessity Score = BMQ-HAART total Necessity score > 24, Low Necessity Score = BMQ-HAART total Necessity score $\leq$ 24.
[b]High Concerns Score = BMQ-HAART total Concerns score >33, Low Concerns Score = BMQ-HAART total Concerns score $\leq$ 33.
[c]Age at ART initiation, type of ART regimen, occupation and type of care were excluded from the multivariable model due to collinearity concerns.
[d]Collapsed into a binary variable due to small numbers in some categories.
